# Polyploidy in the Conifer Genus *Juniperus*: An Unexpectedly High Rate

**DOI:** 10.3389/fpls.2019.00676

**Published:** 2019-05-22

**Authors:** Perla Farhat, Oriane Hidalgo, Thierry Robert, Sonja Siljak-Yakovlev, Ilia J. Leitch, Robert P. Adams, Magda Bou Dagher-Kharrat

**Affiliations:** ^1^Laboratoire Biodiversité et Génomique Fonctionnelle, Faculté des Sciences, Université Saint-Joseph, Campus Sciences et Technologies, Beirut, Lebanon; ^2^Ecologie Systématique Evolution, Univ. Paris-Sud, CNRS, AgroParisTech, Université Paris-Saclay, Orsay, France; ^3^Royal Botanic Gardens Kew, Richmond, United Kingdom; ^4^Laboratori de Botànica, Facultat de Farmàcia, Universitat de Barcelona, Unitat Associada CSIC, Barcelona, Spain; ^5^Biology Department, Sorbonne Université, Paris, France; ^6^Biology Department, Baylor University, Waco, TX, United States

**Keywords:** *Juniperus*, gymnosperms, conifers, polyploidy, genome size, flow cytometry

## Abstract

Recent research suggests that the frequency of polyploidy may have been underestimated in gymnosperms. One notable example is in the conifer genus *Juniperus*, where there are already a few reports of polyploids although data are still missing for most species. In this study, we evaluated the extent of polyploidy in *Juniperus* by conducting the first comprehensive screen across nearly all of the genus. Genome size data from fresh material, together with chromosome counts, were used to demonstrate that genome sizes estimated from dried material could be used as reliable proxies to uncover the extent of ploidy diversity across the genus. Our analysis revealed that 16 *Juniperus* taxa were polyploid, with tetraploids and one hexaploid being reported. Furthermore, by analyzing the genome size and chromosome data within a phylogenetic framework we provide the first evidence of possible lineage-specific polyploidizations within the genus. Genome downsizing following polyploidization is moderate, suggesting limited genome restructuring. This study highlights the importance of polyploidy in *Juniperus*, making it the first conifer genus and only the second genus in gymnosperms where polyploidy is frequent. In this sense, *Juniperus* represents an interesting model for investigating the genomic and ecological consequences of polyploidy in conifers.

## Introduction

Polyploidy or whole genome duplication (WGD) is the heritable condition of possessing more than two complete sets of chromosomes (Comai, 2005). Typically, polyploidy arises either as a result of genome duplication within a species (i.e., autopolyploidy), or from hybridization between two different species followed by chromosome doubling (allopolyploidy) (Stebbins, 1947; Comai, 2005). Most of our understanding of the consequences of polyploidy in plants has come from the study of angiosperms, where it has been shown that polyploidization generally causes a dramatic change in genomic structure, dynamics and expression, and cell organization (Tayalé and Parisod, 2013; Van de Peer et al., 2017; Wendel et al., 2018). Indeed, polyploidy is considered to have played a major role in angiosperm evolution (Blanc and Wolfe, 2004; Chen, 2007; Otto, 2007; Soltis and Soltis, 2009).

While polyploidy has been reported to occur across all major taxonomic land plant groups (Barker et al., 2016), it has been estimated to be very frequent in angiosperms with 50–80% of species being polyploid (Masterson, 1994; Otto and Whitton, 2000) and possibly all angiosperms contain at least one WGD in their ancestry (Van de Peer et al., 2017). In contrast, only 5% of all gymnosperms are reported to be polyploid based on chromosome counts (Khoshoo, 1959; Ahuja, 2005; Husband et al., 2013; Rice et al., 2015). Nevertheless, recent analyses of transcriptomic and genomic data (e.g., Li et al., 2015; Guan et al., 2016; Roodt et al., 2017) have suggested that the evolution of gymnosperms was accompanied by several ancient WGD events, including two within conifers, one at the base of Pinaceae (c. 200–342 million years ago) and one at the base of the cupressophytes (including Cupressaceae but excluding Araucaceae) (c. 210–275 million years ago). This highlights the importance of polyploidy in the very early evolution of conifers in contrast to the extreme rarity of this phenomenon among extant species [estimated to be 1.5% based on chromosome counts (Khoshoo, 1959; Husband et al., 2013; Rice et al., 2015)]. The one notable exception to the low frequency of polyploidy in extant gymnosperms is in *Ephedra*, which belongs to the non-coniferous lineage Gnetales. Here, polyploidy has been reported in over 65% of extant *Ephedra* species (Ickert-Bond et al., 2015). In this genus no evidence for any ancient WGDs has been detected in its ancestry (Li et al., 2015).

Conifers comprise the largest group of extant gymnosperms (Christenhusz et al., 2011), and from a phylogenetic perspective, they are divided into two major clades—(i) the Pinaceae and (ii) cupressophytes as they include Cupressaceae which is the most species-rich family (Lu et al., 2014; Ran et al., 2018). Within extant conifers, chromosome counts of all studied wild stands of all genera of Pinaceae are reported to be diploid (2*n* = 2*x* = 24) (Hizume, 1988; Murray, 2013) despite an exceptional genome size variation in some genera, such as *Pinus* L. (34.5–72.0 pg/2C) (Bogunic et al., 2003; Murray et al., 2012).

Similarly, in Cupressaceae, among ca. 91 species studied for their chromosome number to date (Hair, 1968; Murray, 2013), nearly all are diploid (2*n* = 2*x* = 22), with just three natural polyploids reported: *Sequoia sempervirens* is hexaploid with 2*n* = 6*x* = 66 (Ahuja and Neale, 2002; Scott et al., 2016), while *Fitzroya cupressoides* (Molina) I. M. Johnst. (alerce) and *Juniperus thurifera* L. are tetraploid with 2*n* = 4*x* = 44 (Hair, 1968; Romo et al., 2013; Vallès et al., 2015). It is also notable that within *Juniperus*, the study of just three species revealed each had polyploid cytotypes in some populations (Sax and Sax, 1933; Nagano et al., 2007). These findings raise the question of whether polyploidy may be common in this genus and hence whether it has played a more significant role in the evolution of Cupressaceae than previously recognized in gymnosperms as a whole, and in conifers in particular.

In this study, we focused on exploring the prevalence of polyploidy in wild populations of *Juniperus*. With 115 taxa (75 species with 40 varieties; Adams (2014), also see Table 1 for species and varieties), *Juniperus* is the most diverse genus in Cupressaceae and the second most diverse in all conifers after *Pinus* (Farjon, 2010; Romo et al., 2013). *Juniperus* has been shown to be a well-supported monophyletic genus (Mao et al., 2010; Adams and Schwarzbach, 2013; Adams, 2014), that can be divided into three monophyletic sections: (i) section *Caryocedrus*, with one species in the Mediterranean; (ii) sect. *Juniperus*, with 14 species, 12 in East Asia and the Mediterranean, and one with a circumboreal distribution (*Juniperus communis* L.) and one [*J. jackii* (Rehder) R. P. Adams] endemic to North America; and (iii) sect. *Sabina*, with ~60 species distributed in southwestern North America, Asia and the Mediterranean region, with outlier species in Africa and the Canary Islands. The few polyploids in wild populations noted above have all been reported to occur in species belonging to sect. *Sabina*. Both diploid and tetraploid cytotypes have been found in some populations of *J. chinensis* L. (Sax and Sax, 1933; Hall et al., 1973; Zonneveld, 2012) and in some populations of *J. sabina* L. (Siljak-Yakovlev et al., 2010; Farhat et al., 2019). Few sporadic triploid and tetraploid cytotypes have also been found in some ornamental cultivars. *Juniperus thurifera* is the only species reported to be exclusively tetraploid (2*n* = 4*x* = 44 and 40 pg/2C) (Romo et al., 2013; Vallès et al., 2015). More recently, Bou Dagher-Kharrat et al. (2013) showed that *J. foetidissima* Willd. had a very large genome (59.74 pg/2C), c. 3-fold larger than confirmed diploid *Juniperus* species which range from 19.02 to 26.40 pg/2C (Bennett and Leitch, 2012). The exceptional genome size of *J. foetidissima*, suggests this species may be hexaploid (Bou Dagher-Kharrat et al., 2013) but cytogenetic studies are needed to confirm this since genome size alone may be misleading as it can be highly variable between species of the same genus that have the same ploidy level (Ledig, 1998; Morse et al., 2009; Abdel Samad et al., 2014).

**Table 1:** List of the *Juniperus* taxa studied with indication of data collection, type of material, genome size data, and chromosome numbers.

**Section**	***Species***	***var*.**	**Coll #**	**Location source**	**Dried/Fresh**	**Date coll**.	**2C (pg)**	**CV plant (%)**	**CV standard (%)**	**2*n***	**1C*x* (pg)**
*Caryocedrus*	*J. drupacea* Labill.		*Adams 14693*	Turkey	Dried	2015	23.48	7.19	2.44	22^(i)^	11.74
*Juniperus*	*J. brevifolia* (Seub.) Ant.		*Adams 8152*	Azore Islands	Dried	1997	22.28	4.42	2.34	22^(i)^^(ii)^	11.14
	*J. cedrus* Webb and Berthol.		*Adams 11510*	La Palma	Dried	2008	24.70	7.54	2.67	22^(i)^^(ii)^	12.35
	*J. communis* L.		*Adams 9035*	France	Dried	2000	24.48	4.98	3.56	22^(i)^	12.24
	*J. communis* L.		*RBGK 1977-1318*	NA	Fresh	2017	22.28	2.9	2.42	22^(i)^	11.14
	*J. communis*	*charlottensis* R. P. Adams	*Adams 10304*	Canada	Dried	2004	22.32	4.71	2.28	22^(i)^	11.16
	*J. communis*	*depressa* Pursh	*Adams 10940*	New Mexico	Dried	2005	22.13	3.93	2.38	22^(i)^^(ii)^	11.07
	*J. communis*	*hemispherica* (J. and C. Presl) Parl.	*Adams 9045*	Italy	Dried	2000	22.66	3.67	2.09	22^(i)^^(ii)^	11.33
	*J. communis*	*kamchatkensis* R. P. Adams	*Adams 9182-9164*	Denmark	Dried	2000	–	–	–	–	–
	*J. communis*	*kelleyi* R. P. Adams	*Adams 10890*	USA	Dried	2005	22.30	2.86	2.29	22^(i)^	11.15
	*J. communis*	*megistocarpa* Fernald and H. St. John	*Adams 8576*	Quebec	Dried	1998	22.50	4.2	2.44	22^(i)^	11.25
	*J. communis*	*nipponica* (Maxim.) E. H. Wilson	*Adams 8579*	Japan	Dried	1998	21.92	3.97	2.22	22^(i)^	10.96
	*J. communis*	*oblonga* hort. ex Loudon *(=* var*. communis)*	*Adams 8765*	Armenia	Dried	1999	22.29	3.72	2.67	22^(i)^^(ii)^	11.15
	*J. communis*	*saxatilis* Pall.	*Adams 8686*	Japan	Dried	1998	22.87	4.93	2.47	22^(i)^^(ii)^	11.44
	*J. communis*	*saxatilis* Pall.	*Adams 10378*	Spain	Dried	2004	22.30	4.2	3.1	22^(i)^	11.15
	*J. communis*	*saxatilis* Pall.	*Adams 11206*	Norway	Dried	2006	21.82	4.06	2.44	22^(i)^	10.91
	*J. communis*	*saxatilis (sibirica)* Pall.	*Adams 7589*	Mongolia	Dried	1995	23.92	4.22	2.42	22^(i)^	11.96
	*J. deltoides* R. P. Adams		*Adams 14466*	Azerbaijan	Dried	2014	22.87	3.87	2.88	22^(i)^	11.44
	*J. deltoides* R. P. Adams	*spilinanus* (Yalt., Elicin and Terz.) Terz.	*Adams 12064-12065*	Turkey	Dried	2010	22.93	4.34	3.26	22^(i)^	11.47
	*J. formosana* Hayata		*Adams 9071*	Taiwan	Dried	2000	22.31	4.06	2.33	22^(i)^^(ii)^	11.16
	*J. formosana* Hayata		*RBGK 1995-2911*	NA	Fresh	2017	23.03	3.01	2.44	22^(i)^^(ii)^	11.52
	*J. jackii* (Rehder) R. P. Adams		*Adams 10287*	USA	Dried	2004	22.57	3.87	2.44	22^(i)^	11.29
	*J. macrocarpa* Sibth. and Sm.		*Adams 14047*	Turkey	Dried	2013	25.74	4.33	3.2	22^(i)^^(ii)^	12.87
	*J. maderensis* (Menezes) R. P. Adams		*Adams 11497*	Madeira Island	Dried	2008	22.64	5	2.15	22^(i)^	11.32
	*J. mairei* Lemee and H. Leveille		*Adams 6772*	China	Dried	1991	23.16	3.9	2.69	22^(i)^	11.58
	*J. navicularis* Gand.		*Adams 8240*	Portugal	Dried	1997	22.66	4.93	2.5	22^(i)^	11.33
	*J. oxycedrus* L.		*Adams 9039*	France	Dried	2000	23.08	4.98	2.69	22^(i)^^(ii)^	11.54
	*J. oxycedrus*	*badia* H. Gay	*Adams 7795*	Spain	Dried	1996	22.32	3.87	2.93	22^(i)^^(ii)^	11.16
	*J. rigida* Siebold and Zucc.		*Adams 8544*	Japan	Dried	1998	22.31	4.25	2.56	22^(i)^^(ii)^	11.16
	*J. rigida*	*conferta* Parl.	*Adams 8585*	Japan	Dried	1998	21.81	3.43	2.13	22^(i)^	10.91
	*J. taxifolia* Hook. and Arn.		*Adams 8448*	Japan	Dried	1998	22.44	4.79	4.66	22^(i)^^(ii)^	11.22
	*J. taxifolia*	*lutchuensis* (Koidz.) Satake	*Adams 8541*	Japan	Dried	1998	22.04	3.42	2.5	22^(i)^^(ii)^	11.02
	*J. angosturana* R. P. Adams		*Adams 6881*	Mexico	Dried	1991	26.79	3.85	2.51	22^(i)^	13.4
	*J. arizonica* R. P. Adams		*Adams* 14908	USA	Dried	2015	27.64	3.98	2.42	22^(i)^	13.82
	*J. ashei* Buchholz		*Adams 12260*	USA	Dried	2004	25.30	3.24	2.24	22^(i)^^(ii)^	12.65
	*J. barbadensis* L.		*Adams 5368*	St. Lucia	Dried	1996	24.20	2.73	2.35	22^(i)^	12.1
	*J. barbadensis*	*lucayana* Britton	*Adams 11408*	Bahamas	Dried	2008	24.21	3.16	2.67	22^(i)^	12.11
	*J. bermudiana* L.		*Adams 2554*	Bermuda Island	Dried	1995	25.81	3.2	2.7	22^(i)^^(ii)^	12.91
*Sabina*	*J. bermudiana L*.		*RBGK 2011-1530*	NA	Fresh	2017	25.51	3.19	2.71	22^(i)^^(ii)^	12.76
	*J. blancoi* Martinez		*Adams 10258-10259*	Mexico	Dried	2004	24.82	3.97	2.35	22^(i)^	12.41
	*J. blancoi*	*huehuentensis* R. P. Adams, S. Gonzalez, and M. G. Elizondo	*Adams 10247*	Mexico	Dried	2004	24.83	3.95	2.95	22^(i)^	12.42
	*J. blancoi*	*mucronata* (R. P. Adams) Farjon	*Adams 8701*	Mexico	Dried	1998	25.28	3.15	2.37	22^(i)^	12.64
	*J. californica* Carriere		*Adams 8698*	Aizona, USA	Dried	1998	28.16	3.53	2.15	22^(i)^	14.08
	*J. californica* Carriere		*Adams 10154*	California, USA	Dried	2004	28.45	3.16	2.28	22^(i)^	14.23
	*J. carinata* Y. F. YU and V L. K. FU		*Adams 8504*	China	Dried	1998	24.30	3.31	2.21	22^(i)^	12.15
	*J. chinensis* L.		*Adams 8535*	Japan	Dried	1998	47.51	3.38	2.78	44^(i)^/22and 44^(ii)^	11.88
	*J. chinensis*	*procumbens* Sieb. ex Endl.	*Adams 8683*	Japan	Dried	1998	46.77	3.11	3.07	44^(i)^^(ii)^	11.7
	*J. chinensis*	*sargentii* A. Henry	*Adams 8688*	Japan	Dried	1998	49.67	3.77	2.52	44^(i)^/22^(ii)^	12.42
	*J. coahuilensis* (Martinez) Gaussen		*Adams 14814*	Texas, USA	Dried	2016	26.56	5.79	2.88	22^(i)^	13.28
	*J. comitana* Martinez		*Adams 6859*	Mexico	Dried	1991	27.57	5.06	2.8	22^(i)^^(ii)^	13.79
	*J. convallium* Rehder and Wilson		*Adams 6781*	China	Dried	1991	26.29	4.14	2.96	22^(i)^	13.15
	*J. coxii* A. B. Jacks		*Adams 8137*	Chimili Valley, Burma	Dried	1997	50.70	4.51	2.11	44^(i)^	12.68
	*J. davurica* Pallas		*Adams 7253*	Mongolia	Dried	1994	23.99	3.49	2.6	22^(i)^^(ii)^	12
	*J. davurica*	*arenaria* (E. H. Wilson) R. P. Adams	*Adams 10347*	China	Dried	2004	24.30	3.38	2.43	22^(i)^	12.15
	*J. davurica*	*mongolensis* R. P. Adams	*Adams 7254*	Mongolia	Dried	1994	23.80	2.96	2.35	22^(i)^	11.9
	*J. deppeana* Steudel		*Adams 10539*	Mexico	Dried	2005	26.39	3	2.32	22^(i)^	13.2
	*J. deppeana* Steudel		*Adams 10927*	Arizona, USA	Dried	2005	25.93	4.05	2.55	22^(i)^^(ii)^	12.97
	*J. deppeana*	*gamboana* (Mart.) R. P. Adams	*Adams 6869*	Mexico	Dried	1991	26.36	4.1	2.7	22^(i)^	13.18
	*J. deppeana*	*patoniana* (Martinez) Zanoni	*Adams 6837-11904*	Mexico	Dried	1991	–	–	–	–	–
	*J. deppeana*	*robusta* Martinez	*Adams 10255*	Mexico	Dried	2004	25.83	3.01	2.49	22^(i)^	12.92
	*J. deppeana*	*robusta* Martinez	*Adams 10256*	Mexico	Dried	2004	25.76	3.62	2.45	22^(i)^	12.88
	*J. deppeana*	*sperryi* (Correll) R. P. Adams	*Adams 11494*	USA	Dried	2008	25.75	3.91	2.67	22^(i)^	12.88
	*J. deppeana*	*zacatacensis* (Mart.) R. P. Adams	*Adams 10517-10518*	Mexico	Dried	2009	25.80	2.9	2.33	22^(i)^	12.9
	*J. durangensis* Martinez		*Adams 10253-11929*	Mexico	Dried	2009	25.54	3.73	2.58	22^(i)^	12.77
	*J. durangensis*	*topiensis* R. P. Adams and S. Gonzalez	*Adams 11923*	Mexico	Dried	2009	25.64	4.33	2.12	22^(i)^	12.82
	*J. erectopatens* (Cheng and L. K. Fu) R. P. Adams		*Adams 8532-8533-8534*	China	Dried	1998	–	–	–	–	–
	*J. excelsa* M.-Bieb.		*Adams 14742*	Greece	Dried	2015	27.41	4.47	2.27	22^(i)^^(ii)^^(iii)^	13.71
	*J. fargesii* (Rehder and Wils.) Kom.		*Adams 6769*	China	Dried	1991	25.33	3.65	2.27	22^(i)^	12.67
	*J. flaccida* Schlecht.		*Adams 6892*	Mexico	Dried	1991	26.05	3.56	2.37	22^(i)^	13.03
	*J. foetidissima* Willd.		*Adams 14511*	Greece	Dried	2015	71.32	3.56	3.15	66^(i)^^(iii)^	11.89
	*J. foetidissima* Willd.		*Adams* (waiting for assignment)	Lebanon	Fresh	2017	69.71	3.84	3.91	66^(i)^^(iii)^	11.62
	*J. foetidissima* Willd.		*Adams* (waiting for assignment)	Turkey	Fresh	2018	70.7	3.4	3.2	66^(i)^^(iii)^	11.78
	*J. gracilior* Pilger		*Adams 7664*	Dom. Rep.	Dried	1996	24.97	3.15	2.29	22^(i)^	12.49
	*J. gracilior*	*ekmanii* (Florin) R. P. Adams	*Adams 7653*	Haiti	Dried	1996	25.59	4.44	2.6	22^(i)^	12.8
	*J. gracilior*	*urbaniana* (Pilger and Ekman) R. P. Adams	*Adams 12314*	Dom. Rep.	Dried	2009	28.05	4.28	2.42	22^(i)^	14.03
	*J. gracilior*	*saxicola* (Britton and P. Wilson) R. P. Adams	*Adams 5284*	Cuba	Dried	1985	25.55	3.29	2.24	22^(i)^	12.78
	*J. grandis* R. P. Adams		*Adams 11963*	California, USA	Dried	2009	25.81	3.04	2.47	22^(i)^	12.91
	*J. horizontalis* Moench		*Adams 14381*	Canada	Dried	2014	24.64	5.25	3.23	22^(i)^^(ii)^	12.32
	*J. indica* Bertol.		*Adams 8775*	Nepal	Dried	1999	48.81	3.95	2.55	44^(i)^^(ii)^	12.2
	*J. indica* Bertol.		*Adams 12943*	Nepal	Dried	2011	48.07	3.7	2.68	44^(i)^	12.02
	*J. indica* Bertol.		*RBGK 2010-2167*	NA	Fresh	2017	48.85	2.13	2.03	44^(i)^	12.21
	*J. indica*	*caespitosa* Farjon	*Adams 7625-7626*	Nepal	Dried	1995	–	–	–	–	–
	*J. jaliscana* Martinez		*Adams 15491-15492*	Mexico	Dried	1991	29.50	3.41	3.02	22^(i)^	14.75
	*J. komarovii* Florin		*Adams 8518*	China	Dried	1998	24.76	3.55	2.21	22^(i)^	12.38
	*J. maritima* R. P. Adams		*Adams 11056*	Vancouver Island, Canada	Dried	2006	25.17	3.75	2.35	22^(i)^	12.59
	*J. martinezii* Perez de la Rosa		*Adams 14901*	Mexico	Dried	2016	27.31	3.87	3.07	22^(i)^	13.66
	*J. microsperma* (Cheng and L. K. Fu) R. P. Adams		*Adams 8522*	China	Dried	1998	23.66	3.89	1.97	22^(i)^	11.83
	*J. monosperma* (Engelm.) Sarg.		*Adams 10932*	New Mexico	Dried	2005	26.96	4.02	2.12	22^(i)^^(ii)^	13.48
	*J. monticola* Martinez		*Adams 6876*	Mexico	Dried	1991	24.86	4.82	2.15	22^(i)^	12.43
	*J. morrisonicola* Hayata		*Adams 8681*	Taiwan	Dried	1998	46.61	2.8	2.62	44^(i)^	11.65
	*J. occidentalis* Hook.		*Adams 13546*	Oregon, USA	Dried	2012	26.39	3.85	2.03	22^(i)^^(ii)^	13.2
	*J. osteosperma* (Torr.) Little		*Adams 14310*	Utah, USA	Dried	2014	26.87	5.41	3.18	22^(i)^	13.44
	*J. ovata* R. P. Adams		*Adams 12279*	Texas, USA	Dried	2010	25.48	4.95	2.9	22^(i)^	12.74
	*J. phoenicea* L.		*Adams 13813*	Spain	Dried	2013	24.76	4.43	2.98	22^(i)^^(ii)^	12.38
	*J. phoenicea* L.		*RBGK 1996-114*	NA	Fresh	2017	24.86	2.96	2.47	22^(i)^^(ii)^	12.43
	*J. pinchotii* Sudworth		*Adams 10463*	Texas, USA	Dried	2004	26.24	3.3	2.04	22^(i)^	13.12
	*J. pingii* Cheng and Ferre		*Adams 8506*	China	Dried	1998	25.49	3.23	2.16	22^(i)^	12.75
	*J. pingii*	*miehei* Farjon	*Adams 13598*	Tibet	Dried	2000	29.11	5.55	1.9	22^(i)^	14.56
	*J. poblana* (Mart.) R. P. Adams		*Adams 15208-15209*	Mexico	Dried	2016	24.39	3.98	2.75	22^(i)^	12.2
	*J. poblana* (Mart.) R. P. Adams		*Adams 14898*	Nayarit, MX	Dried	2016	26.95	4.42	2.29	22^(i)^	13.48
	*J. poblana* (Mart.) R. P. Adams	*decurrens* R. P. Adams	*Adams 11926*	Durango, Mexico	Dried	2009	–	–	–	–	–
	*J. polycarpos* K. Koch		*Adams 14171*	Azerbaijan	Dried	2013	24.92	4.68	3.09	22^(i)^	12.46
	*J. polycarpos*	*turcomanica* (B. Fedtsch.) R. P. Adams	*Adams 8757*	Turkmenistan	Dried	1999	24.89	2.76	2.4	22^(i)^	12.45
	*J. procera* Hochst. ex. Endl.		*Adams 15222-15223*	Ethiopia	Dried	2016	24.44	4.2	2.46	22^(i)^^(ii)^	12.22
	*J. procera* Hochst. ex. Endl.		*RBGK 2013-277*	NA	Fresh	2017	24.01	3.42	2.34	22^(i)^^(ii)^	12.01
	*J. przewalskii* Kom.		*Adams 6775*	China	Dried	1991	48.90	3.27	2.38	44^(i)^	12.23
	*J. pseudosabina* Fisch., Mey. and Ave-Lall.		*Adams 7808*	Kazakstan	Dried	1996	24.73	3.22	2.32	22^(i)^^(ii)^	12.37
	*J. recurva* Buch.-Ham. ex D. Don.		*Adams 7215*	Nepal	Dried	1993	47.50	2.87	3.78	44^(i)^/22^(ii)^	11.88
	*J. recurva* Buch.-Ham. ex D. Don.		*RBGK 1976-826*	NA	Fresh	2017	49.05	2.55	2.62	44^(i)^/22^(ii)^	12.26
	*J. rushforthiana* R. P. Adams		*Adams 8140*	Bhutan	Dried	1997	49.94	4.52	2.2	44^(i)^	12.49
	*J. sabina* L.		*Adams 14316*	Azerbaijan	Dried	2014	24.65	4.49	2.76	22^(i)^^(ii)^/44(^ii)^	12.33
	*J. sabina* L.	*balkanensis* R. P. Adams and A. N. Tashev	*Adams 14722*	Bulgaria	Dried	2015	46.36	–	–	44^(iiii)^	–
	*J. saltillensis* M. T. Hall		*Adams 6886*	Mexico	Dried	1991	26.32	3.02	2.06	22^(i)^	13.16
	*J. saltuaria* Rehder and Wils.		*Adams 6789*	China	Dried	1991	26.04	4.61	2.34	22^(i)^	13.02
	*J. scopulorum* Sarg.		*Adams 10895*	Utah, USA	Dried	2005	25.10	3.34	2.37	22^(i)^^(ii)^	12.55
	*J. scopulorum* Sarg.		*RBGK 2004-1660*	NA	Fresh	2017	25.89	2.78	2.21	22^(i)^^(ii)^	12.95
	*J. semiglobosa* Regel		*Adams 8210*	Kyrgystan	Dried	1997	26.41	4.4	2.03	22^(i)^	13.21
	*J. semiglobosa* Regel	*jarkendensis* (Kom.) R. P. Adams	*Adams 7820*	China	Dried	1996	24.96	4.06	2.3	22^(i)^	12.48
	*J. semiglobosa* Regel	*talassica* (Lipsky) Silba	*Adams 8220-8221-8222*	Kyrgystan	Dried	1997	27.24	4.8	2.1	22^(i)^	13.62
	*J. seravschanica* Kom.		*Adams 8224*	Kazakhstan	Dried	1997	48.58	2.89	2.99	44^(i)^/22^(ii)^	12.15
	*J. squamata* Buch.-Ham. ex. D. Don in Lambert		*Adams 6796*	China	Dried	1991	48.55	4.86	2.74	44^(i)^	12.14
	*J. squamata*	*meyeri* Rehder (cv.)	*Adams 13547*	China	Dried	2012	46.29	3.88	3.38	44^(i)^	11.57
	*J. squamata*	*wilsonii* (Rehder) R. P. Adams	*Adams 12912*	China	Dried	2012	25.60	9.33	3.14	22^(i)^	12.8
	*J. standleyi* Steyermark		*Adams 15396*	Mexico	Dried	1991	30.30	4.26	2.56	22^(i)^	15.15
	*J. thurifera* L.		*Adams 9452*	Spain	Dried	2001	48.81	3.54	2.56	44^(i)^^(ii)^	12.2
	*J. thurifera* L.		*RBGK 2015-61*	NA	Fresh	2017	47.14	2.59	2.31	44^(i)^^(ii)^	11.79
	*J. thurifera*	*africana* Maire	*Adams 9420*	Morocco	Dried	2001	48.23	3.53	2.16	44^(i)^^(ii)^	12.06
	*J. tibetica* Kom.		*Adams 8516*	China	Dried	1998	48.27	2.9	2.26	44^(i)^	12.07
	*J. tibetica* Kom.		*RBGK 2013-276*	NA	Fresh	2017	49.43	2.96	2.63	44^(i)^	12.36
	*J. tsukusiensis* Masam.		*Adams 8806*	Japan	Dried	1999	23.75	2.94	2.5	22^(i)^	11.88
	*J. tsukusiensis*	*taiwanensis* (R. P. Adams and C-F. Hsieh)	*Adams 9061*	Taiwan	Dried	2000	23.95	3.84	2.67	22^(i)^	11.98
	*J. turbinata* Guss.		*Adams 7202*	Spain	Dried	1993	25.28	4.65	2.8	22^(i)^^(ii)^	12.64
	*J. turbinata* Guss.		*Adams 12397*	Turkey	Dried	2010	26.38	4.14	1.96	22^(i)^	13.19
	*J. uncinata* R. P. Adams		*Adams 7212*	Nepal	Dried	1993	24.51	3.56	2.75	22^(i)^	12.26
	*J. virginiana* L.		*Adams 10231*	Tennessee USA	Dried	2004	24.91	2.49	1.79	22^(i)^^(ii)^	12.46
	*J. virginiana*	*silicicola* (Small) E. Murray	*Adams 11113-11114*	Florida, USA	Dried	2006	24.81	3.53	2.2	22^(i)^	12.41
	*J. virginiana*	*silicicola* (Small) E. Murray	*RBGK 1984-8179*	NA	Fresh	2017	24.66	4.6	3.94	22^(i)^	12.33
	*J. zanonii* R. P. Adams		*Adams 6900*	Mexico	Dried	1991	25.19	3.3	3	22^(i)^	12.6

“*coll #” correspond to the herbarium voucher specimens deposited at Baylor University Herbarium (BAYLU) “Adams #” or to accessions from the living collections of the Royal Botanic Gardens Kew “RBGK #,” Chromosome numbers*

(i) *deduced from genome size data*,

(ii) *retrieved from CCDB*,

(iii) *directly observed in this study*,

(iiii) *from Farhat et al. (2019), CV: coefficient of variation of the 2C values*.

Altogether, these observations suggest that *Juniperus* may have undergone an unusual evolutionary trajectory, involving polyploidization more frequently than encountered in other conifers. This paper takes a first step toward addressing these gaps in our data to fully understand the role that polyploidization has played in the evolutionary history of *Juniperus*. The objective was to assess variation in genome size across the whole genus and use these data as a proxy to estimate ploidy levels. Using classical cytogenetics approaches, we also determined the ploidy level of *J. foetidissima*, which has the biggest genome in this genus. Finally, we used phylogenetically-informed trait evolution modeling approaches to reconstruct ancestral genome sizes for the three main clades of *Juniperus* and identify the occurrence of polyploidization events during the evolution of *Juniperus*.

## Materials and Methods

### Plant Material

The origins of the studied accessions are presented in Table 1. We used Robert P. Adams's worldwide collection of *Juniperus* leaf material, dried in silica gel and kept frozen at −20°C. This material has been stored for years (the oldest sample was collected in 1985). To address its suitability for genome size analysis and ploidy screening, we carried out measurements on both dry and fresh material for a sub-sample of 12 species which were selected to cover as much of the genus diversity at the taxonomic (representatives of sections *Juniperus* and *Sabina*), morphological (needles-like and scale leaves) and cytogenetic (species with different ploidy levels) levels. Fresh leave material was obtained from plants growing in the living collections of the Royal Botanic Gardens, Kew, UK.

### Genome Size Assessments by Flow Cytometry

Nuclear DNA contents of about 3,000 stained nuclei were estimated for each sample with a CyFlowSL Partec flow cytometer (Partec GmbH) following the one-step protocol of Doležel et al. (2007) with minor modifications as described in Clark et al. (2016). We selected *Allium cepa* L., 2C = 34.89 pg (Doležel et al., 1998; Clark et al., 2016) and the “CyStain PI Absolute P kit” buffer (Sysmex UK) as the most appropriate internal calibration standard and nuclei isolation buffer for ploidy screening in *Juniperus*.

### Chromosome Counts

We compiled published *Juniperus* chromosome numbers from the Chromosome Counts Database (CCDB; Rice et al., 2015). New chromosome counts were made for *J. foetidissima* and *J. excelsa* using 3 years old plants cultivated from seed of natural origin (from Turkey), and following Vallès et al. (2015) for protoplast preparation and Chromomycin A3 (CMA, Serva) staining.

### Analyses of Genome Size and Chromosome Number Evolution

Trait evolution was modeled on the phylogenic tree of Adams (2014), pruned to the set of species and varieties with genome size data and made ultrametric with R v.3.2.2 (Team, 2016). However, five taxa with genome size estimates were not represented in the phylogeny and so they were discarded from these analyses [*Juniperus communis* var. *kelleyi* R. P. Adams, *J. deltoides* var. *spilinanus* (Yalt., Elicin and Terz.) Terz, *J. durangensis* var. *topiensis* R. P. Adams and S. Gonzalez, *J. poblana* var. *decurrens* R. P. Adams, *J. semiglobosa* var. *talassica* (Lipsky) Silba)]. The inference of ancestral genome size values was based on monoploid GS (1C*x*-values) *sensu* Greilhuber et al. (2005). Ancestral 1C*x*-values were reconstructed under ML using the “fastAnc” command and mapped onto the phylogeny with the “contMap” command of the Phytools package of R (Revell, 2012).

We used ChromEvol v.2 (Glick and Mayrose, 2014) to infer ancestral haploid (*n*) chromosome numbers in *Juniperus*. This program implements a series of likelihood models to infer duplication events, chromosome gains/losses and demi-duplications at ancestral nodes. The model that best fitted the data set was chosen under the Akaike information criterion (AIC) using default parameters.

## Results

### Genome Size Diversity

**Figure 1:**
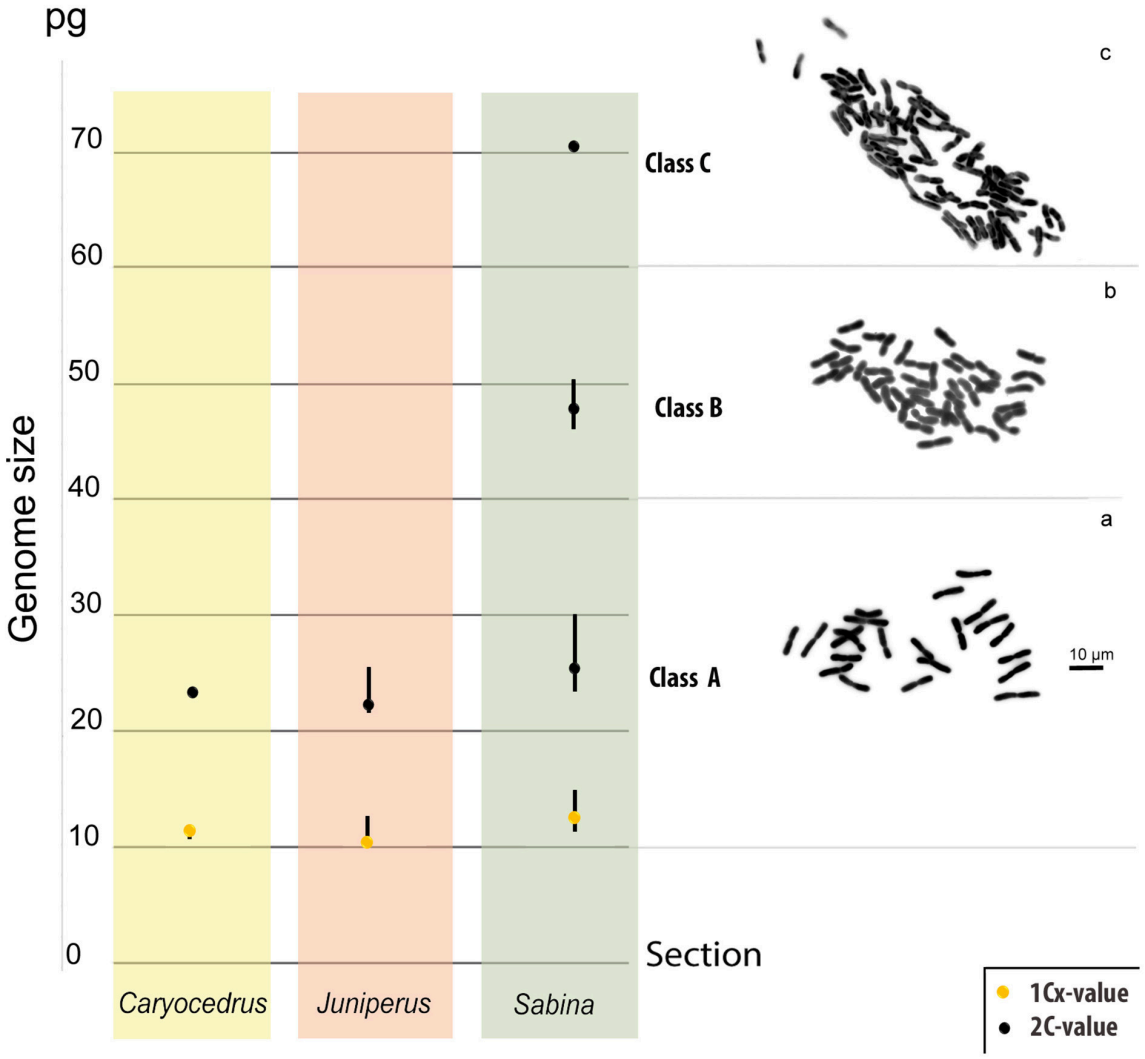
Genome size (2C-values, represented by black dots) classes in *Juniperus* and their unequivocal relationship with the chromosome number. Class A represents the range of genome sizes for all diploid species confirmed by published chromosome numbers. Class B represents the range of genome sizes for all tetraploid species confirmed by published chromosome number. Class C represents the genome size of the only hexaploid species so far reported (i.e., *J. foetidissima*). **(A)** Chromosomes of the diploid *J. excelsa* (our data); **(B)** chromosomes of the tetraploid *J. thurifera* (reproduced from Vallès et al., 2015), and **(C)** Chromosomes of *J. foetidissima* confirming its hexaploid status (our data). Monoploid genome size (1C*x*-values, represented by yellow dots) of the three sections were also illustrated.

Genome sizes were assessed for 111 *Juniperus* species and varieties (Table 1), representing 96.5% of taxonomic diversity. Low differences were found between values obtained with dried and fresh material for the 12 species analyzed using both types of leaf material. Differences varied around zero with six positive (minimum = 0.6%, maximum = 9.8% and mean difference = 3.1%) and six negative percentages (minimum = −0.42%, maximum = −3.16% and mean difference = −2.15%). Overall, the genome size estimates for *Juniperus* ranged 3.2-fold (from 21.81 to 70.58 pg/2C) but they were seen to be distributed into three non-overlapping classes (Figure 1), class A: 21.81–30.3 pg/2C, B: 46.29–50.7 pg/2C, and C: 70.58 pg/2C.

### Ploidy Levels Inferred From Genome Size Data

We gathered chromosome number data from the CCDB for 41 *Juniperus* species and varieties (Table 1). In addition, we made the first chromosome counts for *J. excels*—a diploid with 2*n* = 22, and *J. foetidissima*—a hexaploid with 2*n* = 66 (Figures 1A,C, respectively). Ploidy levels based on chromosome numbers agreed with those inferred from genome size for all but two taxa, suggesting a strong correlation between genome size, ploidy level and chromosome number. Genome size values of class A corresponded to diploids with 2*n* = 2*x* = 22, class B to tetraploids with 2*n* = 4*x* = 44 and class C to hexaploids with 2*n* = 6*x* = 66 (Table 1; Figure 1). The two exceptions were *J. seravschanica* Kom. and *J. chinensis* var*. sargentii* A. Henry, which were both reported to be diploid in the CCDB but had genome size estimates indicating the samples analyzed here were tetraploid. We thus considered these taxa to have two cytotypes, as previously established for *J. chinensis* and *J. sabina* (Table 1).

### Evolution of Chromosome Numbers

**Figure 2:**
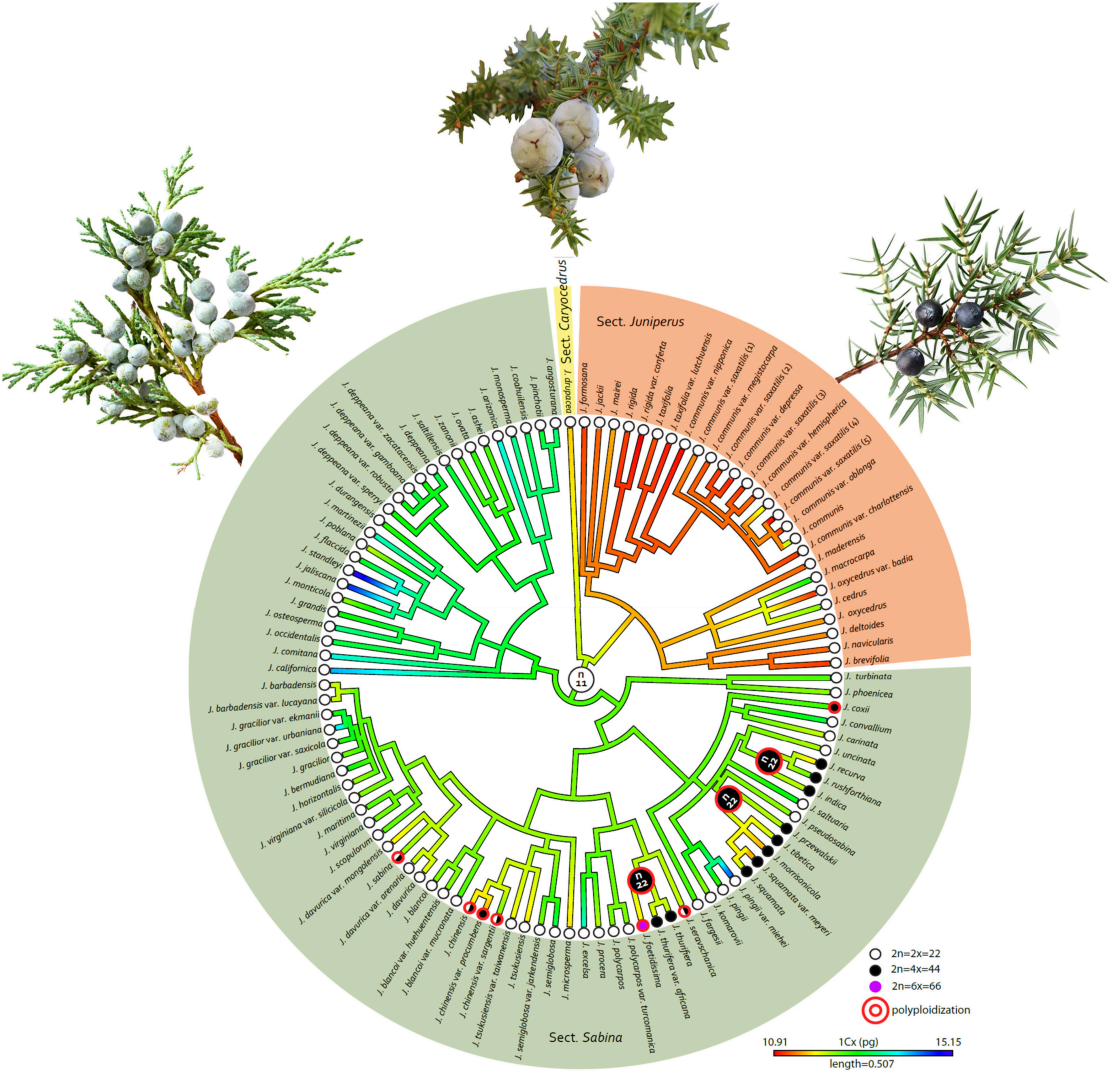
Ancestral state reconstruction of genome size (1C*x*/pg) and chromosome number (n) on a phylogenetic tree of *Juniperus* reconstructed using Bayesian approaches (Adams, 2014). An illustration of the leaf shape for each section is represent by: (a) *J. drupacea* (sect. *Caryocedrus*); (b) *J. communis* (sect. *Juniperus*); (c) *J. excelsa* (sect. *Sabina*).

The best-fitting model in ChromEvol to explain the evolution of chromosome numbers in *Juniperus* was the CONST_RATE model (Supplementary Table S1), suggesting that polyploidy is the predominant mode of chromosome evolution in *Juniperus*. The ancestor of the whole genus was inferred to be diploid, with *n* = 11. It is noted that the polyploids were exclusively restricted to sect. *Sabina* (Figure 2). Three lineage-specific polyploidization events leading to tetraploidy were detected in the ancestors of the clades giving rise to (i) *J. recurva, J. rushforthiana, J. indica*, (ii) *J. preswalskii, J. tibetica, J. morrisonicola, J. squamata*, and (iii) *J. thurifera, J. foetidissima* (Figure 2). A further gain of 22 chromosomes was inferred in the lineage giving rise to the hexaploid *J. foetidissima*. Six species-specific or within-species polyploidization events (i.e., cytotypes) were found in *J. coxii, J. sevaschanica, J. chinensis, J. chinensis* var. *procumbens, J. chinensis* var. *sargentii* and *J. sabina*, all of which contained both diploid and tetraploid individuals (Figure 2).

### Evolution of Genome Size

Beside the genome size variation explained by chromosome number difference, a small variation at the 1C*x*-level was detected between ploidy levels. In addition, the distribution of 1C*x*-values across *Juniperus* presented in Figures 1, 2 showed an ancestral genome size of 12.37 pg for the whole genus and overall larger values in species belonging to sect. *Sabina* (mean 1C*x* 12.7 pg, ancestral 1C*x* 12.64 pg) compared with those of sect. *Caryocedrus* (mean 1C*x* 11.74 pg, ancestral 1C*x* 12.15 pg) and sect. *Juniperus* (mean 1C*x* 11.38 pg, ancestral 1C*x* 11.59 pg). Nevertheless, decreases in 1C*x*-values were observed in several taxa from sect. *Sabina*, including some –but not all– polyploids. Polyploid taxa showed limited 1C*x* variation relative to the value inferred for their most recent ancestors, with a maximum 1C*x* downsizing of 5.70% for *J. squamata* var*. meyeri*, and a maximum 1C*x* upsizing of 1.71% in *J. rushfortiana* (Supplementary Table S2).

## Discussion

### Reliability of Genome Size Estimates From Desiccated Leaf Material of *Juniperus*

Over the years considerable attention has focused on exploring the suitability of dried plant material for genome size and ploidy level analysis, especially given the challenges of collecting and analyzing fresh material from plants growing in remote locations. Dried material has certainly shown to be suitable for ploidy level analysis in many vascular plants (Suda and Trávníček, 2006; Schönswetter et al., 2007; Suda et al., 2007; Popp et al., 2008; Krejčíková et al., 2013; Wang and Yang, 2016). Nevertheless, the quality of data generated by flow cytometry using dried material has been shown to differ between species, buffers (Bainard et al., 2011) and type of desiccation used (Šmarda et al., 2005; Šmarda and Stančík, 2006; Suda and Trávníček, 2006) and it is now generally accepted that while desiccated material is suitable for ploidy level analysis, it is usually not reliable enough for accurate genome size estimations.

In contrast to these previous studies, our analyses of *Juniperus* showed that leaves dried in silica gel and stored continuously at −20°C are suitable for genome size estimations using flow cytometry, giving reasonable data quality (i.e., mean %CV = 3.9, S.D. = 0.96). This was supported by comparisons of 2C-values estimated for the same species from dried and fresh material where low differences between the two variances were found in the 12 species analyzed. We are thus confident that the genome size data generated from the desiccated material analyzed here are reliable and hence suitable for exploring genome size [but there might be a slight shift in “absolute” genome sizes (9.8% at maximum)] and ploidy diversity and evolution across *Juniperus*. Our results broadly agree with Bainard et al. (2011) who found that leaves desiccated immediately in the field using silica gel, was one of the most promising conservation methods, yielding reasonable quality flow cytometry peaks for some species.

### Variability in Genome Size and Polyploidy in *Juniperus*

This study showed that junipers are characterized by possessing large genomes (mean genome size for diploid taxa = 25 pg/2C) with extensive variation between species (ranging 3.2-fold from 21.81 to 71.32 pg/2C). This large variation perfectly correspond to known ploidy levels (2*x* – 6*x*), while the variation in 1C*x* is only 1.38-fold. The data considerably extend our knowledge of genome sizes in *Juniperus* which was previously based on data for just 19 species (Bennett and Leitch, 2012). They also show *Juniperus* now has the largest range in genome size so far reported for any conifer genus.

There are three main mechanisms which can lead to variation in genome size; (i) rapid loss or expansion of transposable and/or other repetitive elements, (ii) loss or gain of chromosomes (aneuploidy and dysploidy), and (iii) polyploidization, possibly followed by genome downsizing (Ramsey and Schemske, 1998; Leitch and Bennett, 2004; Greilhuber et al., 2005; Morse et al., 2009). While in *Pinus* the high variability in genome size (34.50–72.00 pg/2C; Murray et al., 2012) has been shown to be mainly driven by variation in copy numbers of repeats, such as retrotransposable elements (Morse et al., 2009; Kovach et al., 2010; Nystedt et al., 2013), in *Juniperus*, our data indicate that most of the variation in genome size is due to variation in ploidy levels. This does not exclude the occurrence of limited genome size variation within each ploidy level, but based on the data presented, it is relatively small, ranging just 1.4-fold in diploids (95 taxa) and 1.1-fold in tetraploids (15 taxa). The source of this variation is still unclear but likely to represent variation in repeat content since, to date, there have been no reports of aneuploidy in the genus (Murray, 2013).

Among the 111 taxa analyzed, just two (*J. chinensis* var. *sargentii* and *J. seravschanica*) showed a discrepancy between the chromosome number reported in the CCDB and the ploidy level estimated from the genome size data obtained here. This could be due to a technical error, such as misidentification of the species used for counting chromosomes and such an explanation is possible for *J. seravschanica*, where the synonym taxa *J. macropoda* Boiss. has been used to determine the ploidy level (Rice et al., 2015). Nevertheless, these exceptions could also be explained by the existence of intra-specific variability in ploidy levels (= cytotype diversity), a well-documented phenomenon encountered in many land plant lineages, especially in angiosperms and ferns (Husband et al., 2013). In contrast, cytotype diversity is rarely reported in gymnosperms, with *Ephedra* being the only genus where it occurs extensively (>50% of species have >1 cytotype—Ickert-Bond et al., 2015). Prior to this study, natural intraspecific variation in ploidy level in *Juniperus* had only been reported in a few species including in *J. chinensis* (2*x*, 4*x*) (Sax and Sax, 1933; Hall et al., 1973) and *J. sabina* (2*x*, 4*x*) (Siljak-Yakovlev et al., 2010; Farhat et al., 2019).

In view of these previous studies, the results presented here are striking—revealing a much higher frequency of polyploidy in *Juniperus* than hitherto detected, with 15% of taxa being tetraploid, and the discovery of an hexaploid (*J. foetidissima*), which is only the second hexaploid to be found in conifers. In addition, the use of ChromEvol to infer the evolution of chromosome numbers across the phylogeny of *Juniperus* suggests that there have been an unexpectedly high number of polyploidization events throughout its evolutionary history compared with other gymnosperm lineages (except *Ephedra*). Such a result suggests that mechanisms that promote polyploidization and/or the evolutionary success of polyploid species have occurred at a much higher frequency in *Juniperus* than in other conifers, and even in gymnosperms in general, apart from *Ephedra*. It is also worth noting that only one individual was analyzed for most taxa in this study. It is therefore possible that our data underestimate the importance of polyploidization in *Juniperus* as additional intraspecific ploidy diversity may well be uncovered when more individuals are analyzed, as already seen in *J. sabina* and *J. chinensis*.

### Genome Size Evolution and Ploidy Levels of *Juniper* Ancestors

Studies exploring the evolution of genome size diversity across different land plant groups, have uncovered contrasting dynamics in genome size fluctuations throughout their evolution (Bainard and Villarreal, 2013; Clark et al., 2016; Soltis et al., 2018). Now that genome size data are available for almost every recognized taxa of *Juniperus* and that ploidy levels can be inferred given the robust relationship with genome size (Figure 1), the reconstruction of the ancestral genome size within this genus and inferred ancestral ploidy level is highly instructive. Indeed, apart from *Pinus* (Grotkopp et al., 2004), our study is the first to reconstruct ancestral genome size within a species-rich genus for any gymnosperm. Our analysis revealed that the ancestral ploidy level for *Juniperus* was diploid with an estimated genome size of 12.37 pg/1C, which fits within the range of 9–12.38 pg/1C inferred by Burleigh et al. (2012), based on a sampling including only two *Juniperus* species amongst 165 gymnosperm species.

Within the genus, we found evidence suggesting that fluctuations in genome size, both upsizing and downsizing, independent of polyploidy, have taken place during evolution, as also found in *Pinus* (Grotkopp et al., 2004) and across other gymnosperm lineages as well (Burleigh et al., 2012). However, while, in most other gymnosperm genera the shifts in genome size are likely to be driven by changes in the abundance of repetitive DNA (Nystedt et al., 2013; De La Torre et al., 2014), in *Juniperus* the large shifts in genome size are associated with polyploidization events, with a minimum of 10 such events predicted from our analyses (Figure 2). Whether the occurrence and frequency of polyploidy, which was seen to be restricted to sect. *Sabina*, contributes to the higher number of species in this section (c. 60 species) compared with the other two sections of *Juniperus* (sect. *Juniperus* = c. 13 species, sect. *Caryocedrus* = one species) is unclear, although previous studies pointing to higher diversification rates in some angiosperm lineages following polyploidy suggest this is possible (Wood et al., 2009; Landis et al., 2018).

Concerning the origin of the hexaploid, *J. foetidissima*, there are several possible pathways. It could have arisen from a triploid ancestor following one step. If so, then there are two possible routes; (i) fertilization between two unreduced triploid gametes of a triploid ancestor, or (ii) somatic doubling of a triploid, giving rise directly to the hexaploid. Alternatively, it could have arisen following two WGD events (two steps) as envisaged for the hexaploid *Sequoia sempervirens* (Scott et al., 2016). The first step being a WGD event either *via* autopolyploidy or allopolyploidy leading to the formation of a tetraploid with *n* = 2*x*, followed by hybridization with a diploid (*n* = *x*) leading to a triploid. The second step involves a WGD giving rise to a hexaploid. The reports of sporadic triploid *Juniperus* individuals indicate that triploids can indeed form (Hall et al., 1973). However, yet another possibility is that the origin of *J. foetidissima* does not involve a triploid, but instead arose from hybridization between an unreduced gamete from a tetraploid (4*x*) with either (a) a reduced gamete from another tetraploid (2*x*) or (b) an unreduced gamete from a diploid (2*x*). Currently, there is no information about the genomic makeup of *J. foetidissima* to know whether it is an auto- or allo-polyploid, or its mode of origin.

### Why Is Polyploidy More Common in *Juniperus* Than Other Conifers?

The success of hexaploid *Sequoia sempervirens* and polyploid *Ephedra* species (4*x* – 8*x*), has been partially attributed to their capacity for vegetative propagation (Scott et al., 2016; Wu et al., 2016) and this may also contribute to the survival of polyploid *Juniperus* species as there is evidence that they too have the capacity for vegetative propagation [e.g., in *J. sabina* and *J. communis* (Houle and Babeux, 1994; Ronnenberg, 2005; Wesche et al., 2005; Tylkowski, 2010)]. Furthermore, the extreme longevity has been suggested to be another factor contributing to the success of polyploidy in *S. sempervirens* (Scott et al., 2016), and since *Juniperus* has been classified as long-lived (Ward, 1982; Gauquelin et al., 2012) this may also help the survival of polyploids, enabling them to become established.

Here we propose a novel hypothesis that may also contribute to higher frequency of polyploidy revealed in *Juniperus—*this is the high frequency of sympatry between juniper species. In contrast to most of the conifers, the geographical ranges of *Juniperus* species overlap considerably which opens up lots of opportunities for natural hybridization between species. For example, in Spain, hybrids between *J. thurifera* × *J. sabina* and *J. thurifera* × *J. phoenicea* and *J. sabina* × *J. phoenicea* in sympatry have been described (Rojo and Díaz, 2006, 2009; Rojo and Uribe-Echebarría, 2008). More recently, Adams et al. (2016) suggested that an ancient hybridization between *J. thurifera* and *J. sabina* gave rise to *J. sabina* var. *balkanensis*. Juniper hybrids are also common in North America between closely related species in areas of sympatry [e.g., between *J. virginiana* L. and *J. horizontalis* Moench*, J. osteosperma* Hook and *J. occidentalis* Torr. Little, *J. virginiana* var*. silicicola*, and *J. bermudiana* (Vasek, 1966; Palma-Otal et al., 1983; Adams and Kistler, 1991; Adams and Wingate, 2008; Adams, 2014)].

Even though the sympatry is a *sine qua non* condition for natural hybridization, there are few cases of conifers occurring in sympatry that do hybridize without giving rise to polyploids: e.g., *Pinus taeda* and *P. echinata* (Edwards-Burke et al., 1997). Furthermore, induced hybridization like for *Cedrus* species (Fady et al., 2003) produced only homoploids. Cases of unreduced gamete production were documented in Cupressaceae (Pichot and El Maâtaoui, 2000) and Ephedraceae (Wu et al., 2016). This ability to produce unreduced gametes may be the explanation for polyploidisation in *Juniperus*.

On the other hand, the genomic shock arising from hybridization can often be ameliorated by WGD and subsequent diploidization as it was shown in angiosperms (Hegarty et al., 2006). Given the high frequency of hybrid formation in *Juniperus*, and assuming that similar levels of genomic shock following hybridization also occur here, as in angiosperms, then it is possible to envisage that polyploidy may offer one potential solution to these genomic challenges, tipping the balance toward their survival in the wild. Clearly, studies are now needed at the molecular level to provide insights into whether our understanding of the genomic consequences of hybridization and polyploidization in angiosperms is also applicable to the growing list of gymnosperm polyploids.

## Conclusion

Polyploidy or whole genome duplication is rare in conifers. The lack of studies on polyploidy within *Juniperus* prompted the present study, in which the ploidy level of 96.5% of the genus was screened in order to explore the extent of polyploidy across the genus. Silica gel-dried leaves of *Juniperus* were found to be highly suitable for genome size measurements using flow cytometry. This study uncovered a relatively high number of polyploidization events (at least 10) in *Juniperus*, compared to other conifers, and revealed that at least 15% of *Juniperus* taxa are tetraploids. In addition, we used both chromosome and genome size data to validate the presence of the only hexaploid in *Juniperus* (*J. foetidissima)* so far reported, and only the second hexaploid found in conifers (after *Sequoia sempervirens*). An analysis of the phylogenetic distribution of polyploids across *Juniperus* showed they were restricted to sect. *Sabina* and that three clades are exclusively made of polyploids (one including the hexaploid *J. foetidissima*), providing the first evidence of possible lineage-specific polyploidizations in the genus.

Overall, it seems clear that *Juniperus* is exceptional within conifers, and represents a second genus within gymnosperms where polyploidy is common. We propose that *Juniperus* should be considered to be a highly relevant model for studying polyploidization mechanisms and pathways in conifers, and comparisons with *Ephedra* will provide a comprehensive understanding of the evolutionary dynamics and consequences of polyploidy in gymnosperms.

## Author Contributions

MB designed the study. RA provided the *Juniperus* material. PF and OH carried out the flow cytometry measurements and analyzed the data. PF and SS-Y determined the chromosome numbers. PF wrote a first draft of the manuscript that was further critically reviewed by MB, RA, OH, SS-Y, IL, TR.

### Conflict of Interest Statement

The authors declare that the research was conducted in the absence of any commercial or financial relationships that could be construed as a potential conflict of interest.
