# Supplementary material for: Polyploidy in the Conifer Genus Juniperus: An Unexpectedly High Rate
Source: Front Plant Sci. 2019 May 22;10:676. doi: 10.3389/fpls.2019.00676 (PMC6541006; doi:10.3389/fpls.2019.00676)
Supplement: Supplementary file 1 [file Table_1.DOCX]

Supplementary Material

Supplementary Table S1: Model summary of the ChromEvol analysis. Underlined value indicates the best AIC fitting model.

| Model | Log-likelihood | AIC |
| --- | --- | --- |
| CONST_RATE | -29,27 | 64,53 |
| CONST_RATE_DEMI | -32,79 | 71,59 |
| CONST_RATE_DEMI_EST | -29,27 | 66,54 |
| CONST_RATE_NO_DUPL | -215,2 | 434,3 |
| LINEAR_RATE | -29,27 | 68,53 |
| LINEAR_RATE_DEMI | -32,80 | 75,60 |
| LINEAR_RATE_DEMI_EST | -29,27 | 70,53 |
| LINEAR_RATE_NO_DUPL | -215,2 | 438,3 |

Supplementary Table S2: Variation of 1C*x* in polyploid *Juniperus* taxa relative to the ancestral genome size reconstructed at the base of the polyploid lineage. For isolated polyploid species (indicated with *), variation of 1C*x* in polyploid *Juniperus* taxa relative to the value interred for their most recent ancestor. Underlined values indicate minimum and maximum 1Cx variation.

| Taxon | 1C*x* ancestor  (pg) | 1C*x* taxon  (pg) | 1C*x* variation  (%) |
| --- | --- | --- | --- |
| *J. chinensis* var. p*rocumbens** | 11,86 | 11,69 | -1,43 |
| *J. coxii** | 12,52 | 12,68 | 1,28 |
| *J. foetidissima* | 12,11 | 11,89 | -1,82 |
| *J. indica* | 12,28 | 12,2 | -0,65 |
| *J. morrisonicola* | 12,27 | 11,65 | -5,05 |
| *J. przewalskii* | 12,27 | 12,23 | -0,33 |
| *J. recurva* | 12,28 | 11,88 | -3,26 |
| *J. rushfortiana* | 12,28 | 12,49 | 1,71 |
| *J. squamata* | 12,27 | 12,14 | -1,06 |
| *J. squamata* var. *meyeri* | 12,27 | 11,57 | -5,70 |
| *J. thurifera* | 12,11 | 12,2 | 0,74 |
| *J. thurifera* var. *africana* | 12,11 | 12,06 | -0,41 |
| *J. tibetica* | 12,27 | 12,07 | -1,63 |
